# *Plasmodium falciparum* Infection Status among Children with *Schistosoma* in Sub-Saharan Africa: A Systematic Review and Meta-analysis

**DOI:** 10.1371/journal.pntd.0005193

**Published:** 2016-12-07

**Authors:** Abraham Degarege, Dawit Degarege, Emir Veledar, Berhanu Erko, Mathieu Nacher, Consuelo M. Beck-Sague, Purnima Madhivanan

**Affiliations:** 1 Department of Epidemiology, Robert Stempel College of Public Health & Social Work, Florida International University, Miami, Florida, United States of America; 2 Aklilu Lemma Institute of Pathobiology, Addis Ababa University, Addis Ababa, Ethiopia; 3 Ethiopian Ministry of Health Office, Addis Ababa, Ethiopia; 4 Ecosystemes Amazoniens et Pathologie Tropicale, Université de Guyane, Cayenne, French Guiana; 5 Centre d’Investigation Clinique, CIC INSERM 1424, Centre Hospitalier Andrée Rosemon, Cayenne, French Guiana; 6 Department of Health Promotion and Disease Prevention, Robert Stempel College of Public Health & Social Work, Florida International University, Miami, Florida, United States of America; 7 Public Health Research Institute of India, Mysore, India; London School of Hygiene and Tropical Medicine, UNITED KINGDOM

## Abstract

**Background:**

It has been suggested that *Schistosoma* infection may be associated with *Plasmodium falciparum* infection or related reduction in haemoglobin level, but the nature of this interaction remains unclear. This systematic review synthesized evidence on the relationship of *S*. *haematobium* or *S*. *mansoni* infection with the occurrence of *P*. *falciparum* malaria, *Plasmodium* density and related reduction in haemoglobin level among children in sub-Saharan Africa (SSA).

**Methodology/Principal findings:**

A systematic review in according with PRISMA guidelines was conducted. All published articles available in PubMed, Embase, Cochrane library and CINAHL databases before May 20, 2015 were searched without any limits. Two reviewers independently screened, reviewed and assessed all the studies. Cochrane Q and Moran’s I^2^ were used to assess heterogeneity and the Egger test was used to examine publication bias. The summary odds ratio (OR), summary regression co-efficient (β) and 95% confidence intervals (CI) were estimated using a random-effects model. Out of 2,920 citations screened, 12 articles (five cross-sectional, seven prospective cohort) were eligible to be included in the systematic review and 11 in the meta-analysis. The 12 studies involved 9,337 children in eight SSA countries. Eight studies compared the odds of asymptomatic/uncomplicated *P*. *falciparum* infection, two studies compared the incidence of uncomplicated *P*. *falciparum* infection, six studies compared *P*. *falciparum* density and four studies compared mean haemoglobin level between children infected and uninfected with *S*. *haematobium* or *S*. *mansoni*. Summary estimates of the eight studies based on 6,018 children showed a higher odds of asymptomatic/uncomplicated *P*. *falciparum* infection in children infected with *S*. *mansoni* or *S*. *haematobium* compared to those uninfected with *Schistosoma* (summary OR: 1.82; 95%CI: 1.41, 2.35; I^2^: 52.3%). The increase in odds of asymptomatic/uncomplicated *P*. *falciparum* infection among children infected with *Schistosoma* remained significant when subgroup analysis was conducted for *S*. *haematobium* (summary OR: 1.68; 95%CI: 1.18, 2.41; I^2^: 53.2%) and *S*. *mansoni* (summary OR: 2.15; 95%CI: 1.89, 2.46: I^2^: 0.0%) infection. However, the density of *P*. *falciparum* infection was lower in children co-infected with *S*. *haematobium* compared to those uninfected with *Schistosoma* (summary-β: -0.14; 95% CI: -0.24, -0.01; I^2^: 39.7%). The mean haemoglobin level was higher among children co-infected with *S*. *haematobium* and *P*. *falciparum* than those infected with only *P*. *falciparum* (summary-mean haemoglobin difference: 0.49; 95% CI: 0.04, 0.95; I^2^: 66.4%)

**Conclusions/Significance:**

The current review suggests *S*. *mansoni* or *S*. *haematobium* co-infection may be associated with increased prevalence of asymptomatic/uncomplicated *P*. *falciparum* infection in children, but may protect against high density *P*. *falciparum* infection and related reduction in haemoglobin level.

## Author Summary

A clear understanding of the epidemiology of malaria during *Schistosoma* co-infection is essential to inform decisions on appropriate control strategies for schistosomiasis and malaria in SSA. In this systematic review and meta-analysis, we synthesized evidence on the nature of relationship of *S*. *haematobium* and *S*. *mansoni* infection with the prevalence/incidence of *P*. *falciparum* infection, density of the parasite and related reduction in haemoglobin level among children in SSA. We searched all published articles available in PubMed, Embase, Cochrane library and CINAHL databases before May 20, 2015 without any language restriction. We found five cross-sectional and seven prospective cohort studies eligible to be included in the systematic review, and 11 of these studies were included in the meta-analysis. A summarized analysis of the study findings showed that *S*. *haematobium* and *S*. *mansoni* infection is associated with an increased odds of asymptomatic/uncomplicated *P*. *falciparum* infection. However, density of *P*. *falciparum* infection decreased and haemoglobin level increased during *S*. *haematobium* co-infection.

## Introduction

Malaria and schistosomiasis are common in tropical and sub-tropical areas, causing high burden of morbidity and mortality, particularly in children [1,2]. In 2015, about 214 million people were infected and 438,000 estimated to have died globally due to malaria [1]. Additionally, more than 261 million people required preventive treatment for schistosomiasis and close to 200,000 are estimated to die due to this disease annually [2]. About 90% of the malaria deaths and 90% of those who require treatment for schistosomiasis live in sub-Saharan Africa (SSA), with children being the most affected group [1,2]. *Plasmodium falciparum* (*P*. *falciparum*) is responsible for most malaria cases and deaths due to the disease in SSA [3–5]. Likewise, two schistosome species, *Schistosoma mansoni* (*S*. *mansoni*) and *S*. *haematobium* are responsible for almost all of schistosomiasis cases in SSA [6]. *S*. *mansoni* causes intestinal and hepatic schistosomiasis and *S*. *haematobium* causes urogenital schistosomiasis [6]. Both *Schistosoma* spp. cause inflammation that leads to anaemia, growth stunting or cognitive impairment [6].

Humans infected with *Plasmodium* species that cause malaria can manifest a wide range of symptoms that vary from asymptomatic infection to severe complications resulting in death [6–8]. Severe malaria complications such as cerebral malaria, respiratory failure, acute renal failure or severe anaemia usually occur when unimmune individuals get infected with *P*. *falciparum* [7]. On the other hand, people living in regions where there is stable malaria transmission will usually show common symptoms such as fever, chills, fatigue, malaise when infected with *Plasmodium* spp. [8,9]. Still some immune individuals infected with *Plasmodium* may not develop fever, chills or other acute clinical symptoms of malaria, or may show symptoms intermittently but not severe enough to require attention from a health care provider [8].

*Schistosoma* co-infection can affect the development of *Plasmodium* infection related symptoms by altering the immune function [10]. Distributions of *Plasmodium* and *Schistosoma* species overlap in most of SSA, resulting in high rates of co-infection [11]. Based on the immunological findings in murine models and human subjects, it is hypothesized that there is a down-regulating effect of *Schistosoma* on the immune system of individuals, which in turn, may affect the course of other intracellular infections like *Plasmodium* [10]. However, research on the course of *P*. *falciparum* infection and related outcomes during *Schistosoma* co-infection has generated contradictory findings. While some studies report increased odds of *P*. *falciparum* infections and/or malaria-related complications associated with *S*. *haematobium* or *S*. *mansoni* co-infections [12–14], others reported lower incidence and density of *P*. *falciparum* infection in children with *S*. *haematobium* infection [15,16]. Some studies reported lack of statistically significant association between *S*. *mansoni* or *S*. *haematobium* and the risk of *P*. *falciparum* infection [17,18]. The differences in study design, age groups, and outcomes may have blurred attempts to reach clear conclusions.

In order to reduce adverse impact of schistosomiasis on child health, it is recommended that children living in endemic regions be treated with praziquantel [19]. However, if co-infection with schistosomiasis may reduce children’s susceptibility to the more severe form of *P*. *falciparum* malaria, then such treatment might have unwanted consequences. Thus, a clear understanding of the epidemiology of malaria during *Schistosoma* co-infection is essential to inform decisions on appropriate control strategies for schistosomiasis and malaria in SSA.

Two previous reviews examining helminth and malaria co-infection, partially addressed the issue of *Schistosoma* and malaria co-infection in the general population based on studies conducted in various regions of the world [20,21]. However, the current systematic review and meta-analysis was undertaken to quantify the odds of asymptomatic/uncomplicated *P*. *falciparum* infection, parasite density and *P*. *falciparum* malaria-related reduction in haemoglobin level in *Schistosoma* co-infected children in SSA.

## Methods

The protocol for this systematic review and meta-analysis was conducted following the PRISMA guidelines [22] (S1 Table). It would have been preferable to register this study with PROSPERO [23, 24], an international registry of systematic reviews, from the onset to minimize the potential for reporting bias. However, we did not decide to seek publication until after the review had been conducted. The aim of PROPERO is to minimize reporting bias, particularly if there have been major changes to methods which could potentially introduce biases through increased knowledge of potentially eligible studies leading to the narrowing of objectives or the addition of new outcome measures. Fortunately, we did not make changes on the methods for conducting this review after we had developed the protocol.

### Eligibility criteria

All epidemiological studies except case studies which reported prevalence or incidence of *P*. *falciparum* infection and/or *Plasmodium* density stratified by the presence or absence of *S*. *haematobium* or *S*. *mansoni* infection among children living in SSA were included. Studies that reported immunology of *P*. *falciparum* malaria and *S*. *haematobium* or *S*. *mansoni* co-infection in the general population were also included if they reported the prevalence or incidence of *P*. *falciparum* infection in children separately. Unpublished studies, conference abstracts, protocol, gray literature, review protocols, studies that involved only adults or pregnant women, animal or *in vitro* studies, and studies conducted outside of SSA were excluded after screening the titles and abstracts. Studies were additionally excluded after full text review when children infected with *Schistosoma* were co-infected with soil transmitted helminths or if they lacked epidemiologic data on *P*. *falciparum* and *Schistosoma* co-infection.

### Outcome measures

The primary outcomes were prevalence/incidence of *P*. *falciparum* infection. Asymptomatic/uncomplicated *P*. *falciparum* malaria was defined as microscopic confirmation of the *Plasmodium* parasite in blood without clinical evidence (signs or symptoms) of severe malaria [25]. Seven studies included in this review did not clearly differentiate cases based on the clinical stages of malaria as asymptomatic and/or symptomatic. Although three studies indicated malaria cases as asymptomatic and symptomatic, results on *P*. *falciparum* and *Schistosoma* co-infection was not done on basis of the clinical stages of malaria. Hence, this review did not make any distinction between asymptomatic and uncomplicated *P*. *falciparum* malaria while estimating the nature of association of *Schistosoma* co-infection with *P*. *falciparum* malaria.

Secondary outcomes included asexual stage (ring forms) *Plasmodium* density per microliter of blood, haemoglobin levels per deciliter of blood and anaemia. Anaemia was defined as haemoglobin level below the cut-off values defined by WHO: 11.0 g/dl for children 6–59 months; 11.5 g/dl for children 5–11 years; 12.0 g/dl for children 12–14 years [26]. *S*. *haematobium* and *S*. *mansoni* infections were confirmed in all studies by urine filtration and Kato Katz techniques, respectively.

### Search methods for identification of studies

Two authors (AD and DD) independently conducted a search in Pubmed, Embase, Cochrane Library, CINAHL databases using keywords: malaria OR *Plasmodium* OR “*Plasmodium falciparum*” OR “*Plasmodium vivax*” in combination with helminth OR *Schistosoma* OR “*Schistosoma mansoni*” OR “*Schistosoma haematobium*” (S2 Table) for articles published before May 20, 2015. The search was limited to humans, but no limits were made on language. References of some related reviews [10,11,20,21] and African Online Journals database were also searched for relevant studies. Following exclusion of duplicates; abstracts and titles of 2,149 papers were screened for eligibility criteria and 45 were chosen for full text evaluation. Any discrepancies in the choice of articles being included in the review were resolved by third reviewer adjudication. However, there was a very low degree of discrepancy between the two authors in the choice of articles for the review.

### Data collection

Information about the author, study area, study design, sample size, age range, *Schistosoma* species investigated, prevalence of *P*. *falciparum* and *Schistosoma* co-infection, diagnosis techniques and the main findings on prevalence/incidence and density of *P*. *falciparum* infection and mean haemoglobin level/ prevalence of anaemia were abstracted and entered into an excel sheet by two authors independently. Any discrepancies were resolved by consensus between the two authors. There was a very low degree of discrepancy between the two authors data that were extracted from the articles.

### Quality and bias assessment

Quality and risk of bias of the studies was evaluated using the effective public health practice project [27]. The quality of the studies was assessed on the basis of selection of the study participants, study design, confounder, blinding, data collection methods and withdrawals and drop-outs comparability.

### Study synthesis

Heterogeneity was assessed using Cochrane Q (Chi-square) and Moran’s I^2^ (Inconsistency) using STATA software (Version 11, Texas, USA) [28]. Publication bias was evaluated using a funnel plot and statistical significance was assessed by the Egger test (bias if p<0.1) [29]. Sub-group analyses were conducted for *S*. *mansoni* and *S*. *haematobium*. Odds ratio, relative risk, regression coefficients, mean differences along with the 95% confidence intervals were used as effect measures. When studies did not report 95% CI for mean differences in *Plasmodium* density or haemoglobin level, we estimated it using p-values as suggested by Altman and Bland [30]. The 95% CI for mean difference in haemoglobin level between children co-infected with *S*. *haematobium* and *P*. *falciparum* and those infected with only *P*. *falciparum* for the studies by Deribew *et al*. [31] and Ateba-Ngoa *et al*. [32] was estimated using the mean and standard deviations values. The mean and standard deviation of haemoglobin levels for Ateba-Ngoa *et al*. [32] was estimated from the median and interquartile range based on the formula suggested by Wan *et al*. [33]. A random effects model was used to estimate the summary Mantel-Haenszel odds ratio of *P*. *falciparum* infection, among children infected with *Schistosoma* and those uninfected with *Schistosoma*. A random effects model was also used to estimate the summary regression coefficients and mean differences of *P*. *falciparum* density among children infected with *S*. *haematobium* and those uninfected with *Schistosoma*.

## Results

### Search results and study characteristics

**Fig 1:**
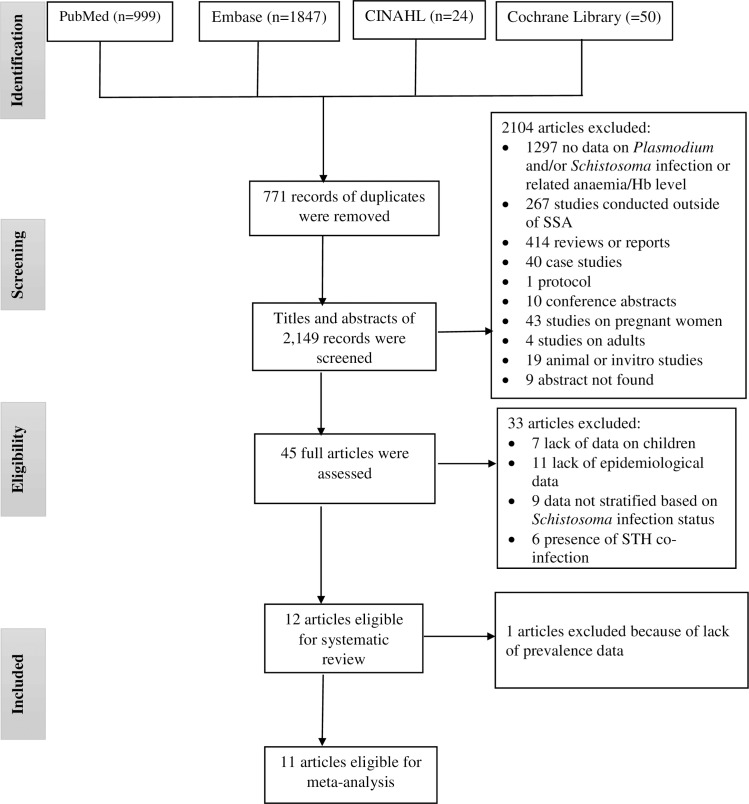
PRISMA diagram. Flow chart for study selection. Hb: Haemoglobin; SSA: sub-Saharan Africa; STH: Soil-transmitted helminths

A total of 2,920 citations were identified from PubMed (n = 999), Embase (n = 1,847), Cochrane library (n = 50) and CINAHL (n = 24), of which 771 articles were found to be duplicates. All relevant articles identified from reviews on malaria and helminth co-infection [10,11,20,21] and African Journal database were similar with the articles found from the four databases. Of the 2,149 articles screened; 2104 articles were excluded after reading the titles and abstracts. Of the 45 full-text articles reviewed, 33 were excluded. A total of 12 articles were considered for the systematic review, of which 11 were included in the meta-analysis (Fig 1).

The characteristics of the 12 studies with 9,337 subjects included in this review are summarized in Table 1. Five studies were cross-sectional and seven were prospective cohorts. Nine studied *S*. *haematobium* and *P*. *falciparum* co-infection, and three studied *S*. *mansoni* and *P*. *falciparum* co-infection. Eight studies compared the odds of asymptomatic/uncomplicated *P*. *falciparum* infection and six studies compared *P*. *falciparum* density between children infected and uninfected with *Schistosoma*. Four studies compared mean haemoglobin level/prevalence of anaemia between children co-infected with *P*. *falciparum* and *Schistosoma* and those infected with only *P*. *falciparum*. Two studies reported uncomplicated *P*. *falciparum* infection, three studies reported both asymptomatic and uncomplicated *P*. *falciparum* infection, but seven studies did not clearly differentiate cases based on the clinical stages of malaria as asymptomatic and/or uncomplicated.

**Table 1:** Characteristics of the included studies.

Reference	Study area (years of the survey)	Sample size (Age range)	Study Design	*Schistosoma* species	Outcomes Reported	Prevalence of ‘*Sh*’ or ‘*Sm*’ & malaria co-infection	The magnitude of outcomes in those co-infected with ‘*Sh*’ or ‘*Sm*’ &*Pf* compared to those with only *Pf*	Diagnostic Techniques
Ateba-Ngoa et al. 2015	Gabon (2011)	125 (6–16 years)	CS	*Sh*	1. *Pf* prevalence 2. Hb level	26%	1. Similar 2. Higher, mean Hb difference:0.7; 95% CI: 0.21, 1.19	Malaria: PCR. *Sh*: Urine filtration, 3 different day samples. Stools not checked
Briand et al. 2005	Senegal (2001 & 2002	523 (3–15 years)	PC	*Sh*	*Pf* density	Not given	Lower in children with light intensity ‘*Sh*’ compared to children without *Schistosoma* (β: -0.34, 95% CI: −0.85, −0.10)	Malaria: microscope. *Sh*: Urine filtration, only1 sample. Other helminth: Kato Katz. Adjusted for age, sex, season and level of exposure
Courtin et al. 2011	Senegal (2003)	234 (6–16 years)	PC	*Sh*	1. *Pf* prevalence 2. *Pf* density	34%	1. Similar, OR: 1.62; 95% CI: 0.94, 2.80 2. Similar	Malaria: microscope. *Sh*: Urine filtration,1 sample. Stools not checked
Deribew et al. 2013	Ethiopia (2008)	387 (6–23 month)	CS	*Sh*	1. *Pf* prevalence 2. Anaemia prevalence 3. Mean Hb	2.84%	1. Higher, OR: 2.8; 95% CI: 1.21, 6.5 2. Higher, OR: 10.12; 95% CI: 1.47, 69.94 3. Similar,	Malaria: microscope. *Sh*: Urine filtration, 1 sample. Stools not checked
Doumbo et al.2014	Mali (2011 & 2012)	616 (3 month to 25 Years)	PC	*Sh*	1. *Pf* prevalence 2. *Pf* risk(hazard ratio) 3. *Pf* density	8.5%	1. Higher, OR: 3.23; 95% CI: 1.76, 5.90 2. Similar 3. Heavy *Sh* negatively associated with *Pf* density but light *Sh* did not associate with *Pf* density	Malaria: microscopy & PCR. *Sh*: Urine filtration. Stools, 2 sample
Florey et al. 2012	Kenya (2005)	223 (8 to 17 years)	CS	*Sh*	*Pf* prevalence	31.8%	Higher, adjusted OR: 1.79; 95% CI: 1.32, 2.44	Malaria: PCR. *Sh*: Urine filtration, 2 samples on different day. Stool not checked. Adjusted for water contact, night activity, bed net use, distance from water
Kabatereine et al. 2011	Uganda (2009 & 2010)	5000 (Mean age = 12.5 years)	CS	*Sm*	*Pf* prevalence	23.5%	Higher, OR: 2.16; 95% CI: 1.89, 2.47	Malaria: microscope. *Sm*: Kato Katz
Lyke et al. 2005	Mali (2002 & 2003)	654 (4–14 years)	PC	*Sh*	1. *Pf* incidence 2. *Pf* density 3. Hb level		1. Lower in children ages 4 to 8 2. Lower in children ages 4 to 8 3. Similar	Malaria: microscope. *Sh*: Urine filtration (*Sh*), 2 to 3 samples. Other helminths: Kato Katz
Mazigo et al. 2010	Tanzania (2009)	400 (8–16 years)	CS	*Sm*	*Pf* prevalence	6%	Higher, OR: 2.1; 95% CI: 1.03, 4.26	Malaria: microscopy. *Sm*: Kato Katz
Lemaitre et al. 2014	Senegal (2001 to 2003)	178 (5–13 years)	PC	*Sh*	*Pf* density		lower in children with light intensity ‘*Sh*’ compared to children without *Schistosoma* (β−0.28; 95% CI: -0.52, -0.04)	Malaria: microscopy. *Sm*: Kato Katz. *Sh*: Urine filtration. Adjusted for Sex, age, village, season
Sangweme et al. 2010	Zimbabwe(2004 & 22005)	485 (6–17 years)	PC	*Sh*	1. *Pf* prevalence 2. *Pf* density 3. mean Hb 4.Anaemia prevalence	18.8%	1. Similar 2. Lower but not significant,Sexual stage ‘*Pf*’ density was higher in children infected than uninfected with ‘*Sh*’ 3. Similar 4. Similar	Malaria: microscopy. *Sh*: Urine filtration, 3 samples on different day. Other helminths: Kato Katz, 3 sample on different day. Only few cases were infected with *Sm*
Sokhna et al. 2004	Senegal (1998)	512 (6–15 years)	PC	*Sm*	*Pf* incidence	13.3%	Higher in children with heavy *Sm* egg load compared to those without helminth (RR: 2.24, 95% CI: 1.20, 4.20)	Malaria: microscope. *Sh*: Urine filtration. Other helminth: Kato Katz

CS: Cross-sectional; CI: Confidence interval; Hb: Haemoglobin; OR: Odds ratio; Pf: *Plasmodium falciparum*; RR: Relative risk; *Sh*: *Schistosoma haematobium*; *Sm*: *Schistosoma manosni*; STH: Soil-transmitted helminths; PC: Prospective cohort; PCR: polymerase chain reaction

Longitudinal studies by Sangweme *et al*. [34], Courtin *et al*. [35] and Doumbo *et al*. [36] did not report incidence, hence the prevalence data reported in these studies during the baseline surveys were used when estimating the summary-odds of *P*. *falciparum* infection in children infected and uninfected with *S*. *haematobium*. However, Sokhna *et al*. [37] reported incidence of *P*. *falciparum* infection in *S*. *mansoni* infected children, the study was thus excluded from the meta-analysis.

### *Schistosoma haematobium* and *Plasmodium falciparum* infection

**Fig 2:**
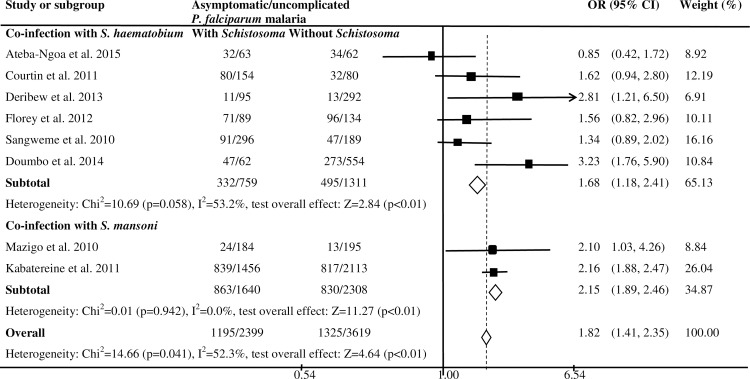
Forest plot showing the difference in the prevalence of asymptomatic/uncomplicated *P*. *falciparum* malaria between children infected with *S*. *haematobium* or *S*. *mansoni* and those not infected with *Schistosoma* in SSA.

Six studies examined the nature of the relationship of *S*. *haematobium* infection with the odds of asymptomatic/uncomplicated *P*. *falciparum* infection (Fig 2). A cross-sectional study in Ethiopia [31] and a prospective cohort study in Mali [36] showed increased odds of asymptomatic/uncomplicated *P*. *falciparum* infection among children infected with *S*. *haematobium* compared to children uninfected with *S*. *haematobium*. A cross-sectional study in Kenya also showed higher odds of asymptomatic/uncomplicated *P*. *falciparum* infection in children infected with *S*. *haematobium* compared to those uninfected with *S*. *haematobium* after adjusting for the effects of water contact, night activity, bednet use and distance from water (adjusted OR: 1.79; 95% CI: 1.32, 2.44) [38]. Although the difference was not statistically significant, prospective cohort studies in Zimbabwe [34] and Senegal [35] showed higher odds of asymptomatic/uncomplicated *P*. *falciparum* infection in children infected with *S*. *haematobium* compared to those who were not infected with *S*. *haematobium*. However, a cross-sectional study in Gabon showed similar odds of asymptomatic/uncomplicated *P*. *falciparum* infection in children infected with *S*. *haematobium* and those uninfected with *S*. *haematobium* [32]. The overall estimates based on six studies showed higher odds of asymptomatic/uncomplicated *P*. *falciparum* infection in children infected with *S*. *haematobium* than those uninfected with *S*. *haematobium* (summary OR: 1.68; 95%CI: 1.18, 2.41; I^2^: 53.2%) [31,32, 34–36, 38]. However, longitudinal studies in Mali showed similar risk of uncomplicated *P*. *falciparum* infection in children infected and uninfected with *S*. *haematobium* [16,36]. Age-stratified analysis of the data in the study by Lyke *et al*. [16], showed association of *S*. *haematobium* infection with fewer episodes of *P*. *falciparum* infection in children of ages 4 to 8 years, but the association was no longer present in children aged 9 to 14 years. Additionally, the study by Doumbo *et al*. [36] showed association of baseline co-infection with *S*. *haematobium* and *P*. *falciparum* with reduced risk of febrile *P*. *falciparum* infection.

### *Schistosoma mansoni* and *Plasmodium falciparum* infection

A cross-sectional study of 5,000 children in Uganda showed higher odds of uncomplicated *P*. *falciparum* infection among children infected than those uninfected with *S*. *mansoni* [39]. Another cross-sectional study in Tanzania documented increased odds of asymptomatic/uncomplicated *P*. *falciparum* malaria among children infected with *S*. *mansoni* than children without *S*. *mansoni* infection [40]. A summary analysis based on the two studies [39,40] showed higher odds of asymptomatic/uncomplicated *P*. *falciparum* infection among children infected than those not infected with *S*. *mansoni* (summary OR: 2.15; 95%CI: 1.89, 2.46; I^2^: 0.0%). Similarly, a prospective cohort study in Senegal showed increased risk of uncomplicated *P*. *falciparum* infection in children with *S*. *mansoni* egg load >1000 eggs/gram as compared to those uninfected with *Schistosoma* (RR: 2.24, 95% CI: 1.20, 4.20) [37]. However, this association was not seen in children with moderate intensity *S*. *mansoni* egg load (100 to 399 eggs/gram). The overall estimates based on eight studies [31, 32, 34–36, 38–40] showed higher odds of asymptomatic/uncomplicated *P*. *falciparum* infection in children infected with *S*. *mansoni* or *S*. *haematobium* as compared to those uninfected with *Schistosoma* (summary OR: 1.82; 95%CI: 1.41, 2.35; I^2^: 52.3%). There was no publication bias detected in the meta-analysis (Egger test = -1.89; p = 0.11), which included eight studies (S1 Fig). Subgroup analysis showed that the increase in odds of asymptomatic/uncomplicated *P*. *falciparum* infection among children infected with *Schistosoma* was significant for studies which used microscopy for the diagnosis of *Plasmodium* infection (summary OR: 1.91; 95%CI: 1.52, 2.40; I^2^: 31.8%) and those conducted in East African region (summary OR: 1.91; 95%CI: 1.51, 2.42; I^2^: 31.7%). However, significant association was not seen between asymptomatic/uncomplicated *P*. *falciparum* infection and *Schistosoma* among studies which used polymerase chain reaction (PCR) for the diagnosis of *Plasmodium* infection and those conducted in the West African region (S2 Fig).

### *Schistosoma* infection and *Plasmodium falciparum* density

**Fig 3:**
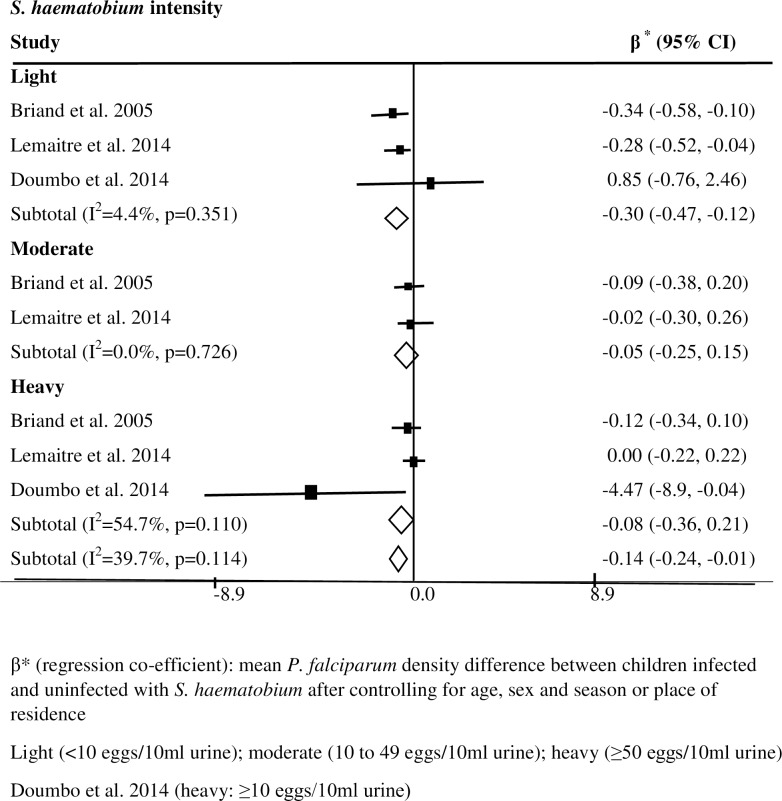
Effect of *S*. *haematobium* infection on *P*. *falciparum* density in children in SSA.

Out of the 12 studies included in this review, six prospective cohort studies examined the effect of *S*. *haematobium* infection on the density of *P*. *falciparum* infection. Among these six studies, two reported lower *P*. *falciparum* density among children with low intensity of *S*. *haematobium* infection (<10 eggs/10ml urine) as compared to those uninfected with *Schistosoma* [15,41]. A study in Mali showed lower *P*. *falciparum* density in children with heavy intensity *S*. *haematobium* infection compared to those uninfected with *Schistosoma* [36]. A summary analysis based on the three studies showed significantly lower *P*. *falciparum* density in children infected with *S*. *haematobium* as compared to those uninfected with *Schistosoma* (summary β = -0.14; 95% CI = -0.24, -0.01; I^2^ = 39.7%) [15, 36, 41]; the difference was greater in children with low intensity *S*. *haematobium* infection (summary β = -0.30; 95% CI = -0.47, -0.12; I^2^ = 4.4%) (Fig 3). A study in Mali also showed significantly lower *P*. *falciparum* density among children aged 4 to 8 years with low-intensity *S*. *haematobium* infection compared to those uninfected with *S*. *haematobium*, however this difference in *P*. *falciparum* density was not significant (p = 0.19) when data were analyzed without stratifying by age and intensity of *S*. *haematobium* infection (5521 *vs*.6761) [16]. Although the difference was not statistically significant, the density of *P*. *falciparum* infection tended to be lower in children infected with *S*. *haematobium* (mean = 1764) compared to those uninfected with *S*. *haematobium* (mean = 2509), irrespective of the intensity of *S*. *haematobium* infection (p = 0.4) [34]. In contrast, a study in Senegal showed lack of significant difference in mean *P*. *falciparum* densities in infected children (mean = 626) compared to those uninfected (mean = 444) with *S*. *haematobium* but data were not stratified based on intensity of *S*. *haematobium* infection (p = 0.56) [35]. Based on a summary analysis of the data in the three studies [16,34,35], *P*. *falciparum* density tended to be lower in children infected with *S*. *haematobium* than those uninfected with the parasite (mean difference = -924.2, 95% CI = -2151.8, 303.4; I^2^ = 0.0). The differences in some of the aforementioned studies in mean *P*. *falciparum* densities among children with moderate or heavy (>10 eggs/10ml urine) intensity of *S*. *haematobium* infection compared to those not infected with the parasite were not significant [15,16,41]. The significant protective effects were largely seen in children with light intensity of *S*. *haematobium* infection.

### Co-infection with *Schistosoma* and *Plasmodium falciparum* and haemoglobin level

**Fig 4:**
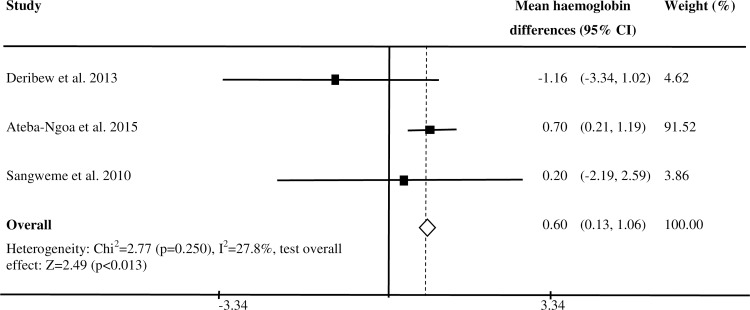
Mean haemoglobin differences between children co-infected with *S*. *haematobium* and asymptomatic/uncomplicated *P*. *falciparum* and those infected with only *P*. *falciparum*.

Out of 12 studies included in this review, four studies reported findings on mean haemoglobin level among children co-infected with *P*. *falciparum* and *S*. *haematobium* and those infected with only *P*. *falciparum*. A study in Gabon reported higher haemoglobin level among children co-infected with *S*. *haematobium* and *P*. *falciparum* than those infected with only *P*. *falciparum* (mean haemoglobin difference = 0.7; 95% CI = 0.21, 1.19) [32]. However, three studies in Mali, Ethiopia and Zimbabwe showed similar mean haemoglobin levels in children co-infected with *S*. *haematobium* and *P*. *falciparum* and those infected with only *P*. *falciparum* [16,31,34]. A summary estimate based on three studies [31, 32,34] showed higher mean haemoglobin level in children co-infected with *S*. *haematobium* and *P*. *falciparum* than those infected with only *P*. *falciparum* (summary mean haemgolobin level difference = 0.49; 95% CI: 0.04, 0.95; I^2^: 66.4%) (Fig 4).Among the four studies, two studies reported the odds of anaemia among children co-infected with *P*. *falciparum* and *S*. *haematobium* and those infected with only *P*. *falciparum*. The study in Ethiopia reported increased odds of anaemia [31], but the study in Zimbabwe [34] reported similar odds of anaemia in children co-infected with *S*. *haematobium* and *P*. *falciparum* as compared to those infected with only *P*. *falciparum*. All the four studies reported similar mean haemoglobin level in children infected with *S*. *haematobium*, and those uninfected with *S*. *haematobium* and *P*. *falciparum* [16, 31, 32,34].

### Quality of the studies

Table 2 summarizes the quality of the studies included in this review in terms of selection bias, study design, confounders, blinding, data collection methods, withdrawals and dropouts. The majority of the studies showed strong quality in data collection methods. The quality of most studies included in this review was moderate in terms of design (prospective cohort), control of selection bias and blinding. Overall rating based on the six criteria showed that two studies were strong quality, six studies were of moderate quality and four studies were of weak quality. None of the studies were excluded from the review because of quality issues.

**Table 2:** Assessment of the quality of the studies included in the review based on Effective Public Health Practice Project: Quality assessment tool for quantitative studies.

No.	Author, Year	SelectionBias	Study Design	Confounders	Blinding	Data Collection Methods	Withdrawals and Drop-Outs	Final Rating
1	Ateba-Ngoaet al., 2015	2	3	3	2	1	NA	2
2	Briand et al. 2005	2	2	1	2	1	1	2
3	Courtin et al. 2011	2	2	3	2	1	3	3
4	Deribew et al. 2013	2	3	3	2	1	NA	3
5	Doumbo et al. 2014	1	2	1	2	1	1	1
6	Florey et al. 2012	3	3	2	2	1	NA	3
7	Kabatereineet al. 2011	2	3	2	2	2	NA	2
8	Lyke et al. 2005	3	2	1	2	1	1	2
9	Mazigo et al. 2010	2	3	3	2	1	NA	3
10	Lemaitre et al. 2014	2	2	1	2	1	3	2
11	Sangweme et al. 2010	1	2	2	2	1	3	2
12	Sokhna et al. 2004	2	2	2	2	1	1	1

1 = strong 2 = moderate 3 = weak NA = not applicable

## Discussion

In the present systematic review of 12 studies based on 9,337 children in eight SSA countries, a meta-analysis of 11 studies confirmed increased odds of asymptomatic/uncomplicated *P*. *falciparum* infection among children infected with *S*. *haematobium* or *S*. *mansoni* as compared to those uninfected with *Schistosoma* in SSA. However, lower *P*. *falciparum* density was associated with *S*. *haematobium* co-infection, particularly when the intensity of *S*. *haematobium* infection was low. The finding of a higher prevalence of asymptomatic/uncomplicated *P*. *falciparum* infection among children infected with *Schistosoma* could be due to the occurrence of common social or environmental factors increasing the susceptibility of individuals to infection with both parasite groups [11]. The increased prevalence of asymptomatic/uncomplicated *P*. *falciparum* infection may result from both increased infection duration and/or increased incidence of the parasite. Increased incidence and infection duration could result from immune responses against chronic *Schistosoma* infection down regulating the effective response against *P*. *falciparum* infection [10]. Hence, *P*. *falciparum* parasites could persist for longer duration in children with chronic *Schistosoma* infection. *P*. *falciparum* sporozoites could have increased survival in liver, which is immunologically affected by *S mansoni* egg granulomas [10]. Children co-infected with *S*. *mansoni* may thus be more susceptible to *P*. *falciparum* infection. Another hypothesis is that anaemia associated with *Schistosoma* infection may cause hyperventilation of carbon dioxide and increased lactates, making the infected individuals more attractive for mosquitoes and thus increasing their risk for *Plasmodium* infection [42].

However, the finding of lower *P*. *falciparum* density among children with low intensity *S*. *haematobium* infection could be due to an imbalance in Th1/Th2 immune response, which might modulate the proportion of cytophylic antibodies that control *Plasmodium* parasitemia [10]. Low intensity *S*. *haematobium* may thus promote an effective Th1 immune response that will also have a protective effect against *P*. *falciparum*. In contrast, increased intensity *S*. *haematobium* infections may enhance a Th2 immune response that alter the protective Th1 response.

Studies included in this review showed varying effects of *Schistosoma* infection on the risk of acquiring *P*. *falciparum* infection [16, 36, 37]. This could be due to varying relationships of *Schistosoma* and *P*. *falciparum* infection based on the age of the host [16] and intensity of *Schistosoma* infection [16, 37] or due to small number of cases with insufficient power to detect statistically significant differences [36]. Indeed, the studies included in this review showed similar risk of acquiring uncomplicated *P*. *falciparum* infection in children infected and uninfected with *S*. *haematobium* when data were analyzed without stratifying by study participants’ ages or *Schistosoma* infection intensity [16, 36, 37]. However, the risk of acquiring uncomplicated *P*. *falciparum* infection and the density of parasitemia varied with the *S*. *haematobium* or *S*. *mansoni* infection status when data were analyzed stratified by age of the study participants or intensity of *Schistosoma* infection. For example, the risk of *P*. *falciparum* infection was higher among children with *S*. *mansoni* egg load >1000 eggs/per gram compared to children without *S*. *mansoni*, but this association was not seen when the analysis was restricted to children with moderate intensity *S*. *mansoni* egg load (100 to 399 eggs/per gram) [37]. Lyke *et al*. [16] showed delayed time to first clinical episode of malaria, fewer episodes, and lower levels of parasitemia in *S*. *haematobium* infected children aged 4 to 8 years compared to children uninfected with *S*. *haematobium*; this protective effect was not seen in children aged 9 to 14 years. Importantly, the decrease in incidence of *P*. *falciparum* infection and density of the parasite as well as the increased time to first clinical *P*. *falciparum* infection was more pronounced in children excreting low numbers of *S*. *haematobium* eggs than those excreting heavy *S*. *haematobium* eggs [16]. Doumbo *et al* [36] found a lower risk of asymptomatic/uncomplicated *P*. *falciparum* infection in children who were co-infected with *S*. *haematobium* and *P*. *falciparum* at baseline, but *S*. *haematobium* mono-infection at baseline (seen only in 23 children) was not significantly associated with protection from *P*. *falciparum* infection.

The summary estimates of the three studies included in this review showed a higher mean haemoglobin level in children co-infected with *S*. *haematobium* and *P*. *falciparum* than in those infected with only *P*. *falciparum* [31, 32, 34]. This result could reflect the immunological effects of *Plasmodium* infection on erythropoiesis and haemoglobin levels through interferon (IFN)-γ and tumor necrosis factor (TNF)-ᾳ activation [43]. Chronic *Schistosoma* infection stimulates anti-inflammatory cytokines interleukin (IL)-10 and Transforming growth factor (TGF)-β, which down-regulate (IFN)-γ and TNF-ᾳ [10]. Hence, *Schistosoma* infection may attenuate the haemoglobin reduction due to immunological effects of *Plasmodium*. However, in some studies included in this review, the findings of a lack of significant difference in haemoglobin levels between children co-infected with *S*. *haematobium* and *P*. *falciparum* and those infected with only *P*. *falciparum* could reflect different clinical stages of *Plasmodium* infection and/or low intensity *Schistosoma* infections. While chronic low *Plasmodium* parasitaemia affect haemoglobin through dyserythropoiesis, the effect of acute initial *Plasmodium* infection on haemoglobin is mainly the result of hemolysis [11]. Moreover, the effect of *Schistosoma* infection on the immunological modulation of *Plasmodium* related dyserythropoiesis could be low when the intensity of infection is light.

### Public health implications

The findings of this review suggest that treatment of children in SSA for schistosomiasis may reduce the risk of asymptomatic/uncomplicated *P*. *falciparum* infection. Hartgers and Yazdanbakhsh, [10] suggested that praziquantel treatment may boost antimalarial immune response by reducing the down-regulating effect of *Schistosoma* [10]. However, the reasons why studies reported lower *Plasmodium* density among children with light [15, 16, 41], heavy [36] or any [34] intensity *S*. *haematobium* infection remains unclear. In addition, the impact of *Schistosoma* infection on risk of acquiring *P*. *falciparum* infection and severity of the disease is poorly understood. Thus, the question of whether the present mass treatment of children living in SSA against schistosomiasis could have some detrimental impact through more severe *P*. *falciparum* infection cannot be definitely answered based on this review. Mass treatment programs generally focus only on the control of schistosomiasis among school-age children in SSA. Since malaria and schistosomiasis frequently co-exist in school-age children and schistosomiasis appears to be associated with increased odds of *P*. *falciparum* infection, collaboration among control programs for both infections and other services for young children might be advantageous.

### Strengths and limitations

This is the first meta-analysis to study association of *P*. *falciparum* and *Schistosoma* co-infection conducted and reported according to the PRISMA guidelines [22]. Studies included in the meta-analysis did not show publication bias. The summary estimates of *P*. *falciparum* density in children infected with *S*. *haematobium* compared with those uninfected with *Schistosoma* were adjusted estimates [15, 36, 41]. However, the present summary results in the meta-analyses of odds of asymptomatic/uncomplicated *P*. *falciparum* infection in children infected with *Schistosoma* were based on crude estimates of individual studies. Only one study provided adjusted estimates of the effect size [38]. Hence, the current summary estimates might have been affected by confounders that can influence the nature of relationship of *Schistosoma* and *P*. *falciparum* in the original studies. In addition, there was a moderate level of bias in selecting the study participants within the studies included in this review. This might have resulted in overestimation of the relationship between *Schistosoma* and asymptomatic/uncomplicated *P*. *falciparum* malaria in the current review. Intervention measures taken against *Schistosoma* or malaria in different regions before the studies could have also altered the nature of relationship between *P*. *falciparum* and *Schistosoma*. This could also have introduced bias into the review. Furthermore, there was a moderate level of heterogeneity among the studies examining association of *P*. *falciparum* and *Schistosoma* co-infection (Moran’s I^2^: 52.3.1%, Cochran’s Q: 14.66, p = 0.041). However, after removing one study [32], the heterogeneity decreased (Moran’s I^2^: 31.1%, Cochran’s Q: 8.66 p = 0.191)] and subgroup analysis further minimized the risk of heterogeneity among studies evaluating the effect of *S*. *mansoni* infection on the odds of *P*. *falciparum* infection (I^2^: 0.0%).

Limitations of the original studies related to the diagnosis of *Plasmodium* and *Schistosoma* infection could also affect the present results [44,45]. In addition, some studies which examined *S*. *haematobium* and *P*. *falciparum* co-infection did not examine the study participants for infection with soil-transmitted helminths. Therefore, children without *S*. *haematobium* infection may have had other helminth infections that could have further confounded the results [46,47]. Moreover, some studies included in this review did not follow WHO criteria to determine classes of intensity of *Schistosoma* infection. This made it difficult to clearly evaluate the potential effect of classes of intensity of *Schistosoma* infection on the current result. Finally, the lack of sufficient data precluded performing a meta-analysis to estimate the effect of *Schistosoma* co-infection on the risk of acquiring *P*. *falciparum* infection and related anaemia. We did not find any studies which examined the relationship between *Schistosoma* and *P*. *vivax* infection or severe malaria that were eligible in the present review.

## Conclusions

The present review suggests that *S*. *mansoni* or *S*. *haematobium* co-infection may increase susceptibility of children for asymptomatic/uncomplicated *P*. *falciparum* infection. However, *S*. *haematobium* co-infection may protect against high *P*. *falciparum* density and related reduction in haemoglobin level. Findings on the effect of *Schistosoma* infection against the risk of *P*. *falciparum* infection are heterogeneous.

## Supporting Information

S1 Table PRISMA checklist. (DOC)

S2 Table Search details for the PubMed database. (DOCX)

S1 Fig Funnel plot. Odds ratio against standard error of odds ratio for eight studies, which compared the prevalence of asymptomatic/uncomplicated *P*. *falciparum* infection between children who were infected and uninfected with *Schistosoma* in SSA.
(TIF)

S2 Fig Forest plot showing the difference in the prevalence of asymptomatic/uncomplicated *P*. *falciparum* malaria between children infected with *S*. *haematobium* or *S*. *mansoni* and those not infected with *Schistosoma* in SSA based on regions and the methods used for the diagnosis of *Plasmodium* infection. (TIF)
