# Supplementary material for: Plasmodium falciparum Infection Status among Children with Schistosoma in Sub-Saharan Africa: A Systematic Review and Meta-analysis
Source: PLoS Negl Trop Dis. 2016 Dec 7;10(12):e0005193. doi: 10.1371/journal.pntd.0005193 (PMC5142807; doi:10.1371/journal.pntd.0005193)
Supplement: S2 Table — (DOCX) [file pntd.0005193.s002.docx]

Supplementary Table 2. Search details for the PubMed

| (("malaria"[MeSH Terms] OR "malaria"[All Fields]) OR ("plasmodium"[MeSH Terms] OR "plasmodium"[All Fields]) OR ("plasmodium falciparum"[MeSH Terms] OR ("plasmodium"[All Fields] AND "falciparum"[All Fields]) OR "plasmodium falciparum"[All Fields]) OR ("plasmodium vivax"[MeSH Terms] OR ("plasmodium"[All Fields] AND "vivax"[All Fields]) OR "plasmodium vivax"[All Fields])) AND (("helminths"[MeSH Terms] OR "helminths"[All Fields] OR "helminth"[All Fields]) OR ("schistosoma"[MeSH Terms] OR "schistosoma"[All Fields]) OR ("schistosoma mansoni"[MeSH Terms] OR ("schistosoma"[All Fields] AND "mansoni"[All Fields]) OR "schistosoma mansoni"[All Fields]) OR ("schistosoma haematobium"[MeSH Terms] OR ("schistosoma"[All Fields] AND "haematobium"[All Fields]) OR "schistosoma haematobium"[All Fields])) AND "humans"[MeSH Terms] |
| --- |
