# Supplementary figures and images for: Plasmodium falciparum Infection Status among Children with Schistosoma in Sub-Saharan Africa: A Systematic Review and Meta-analysis
Source: PLoS Negl Trop Dis. 2016 Dec 7;10(12):e0005193. doi: 10.1371/journal.pntd.0005193 (PMC5142807; doi:10.1371/journal.pntd.0005193)

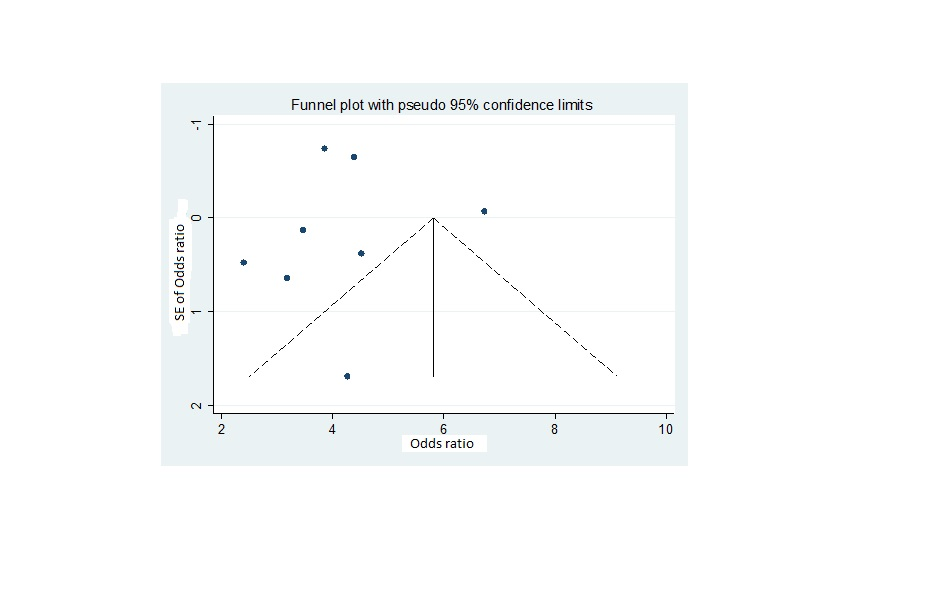

Supplement: S1 Fig — Odds ratio against standard error of odds ratio for eight studies, which compared the prevalence of asymptomatic/uncomplicated P. falciparum infection between children who were infected and uninfected with Schistosoma in SSA. (TIF) [file pntd.0005193.s003.tif]

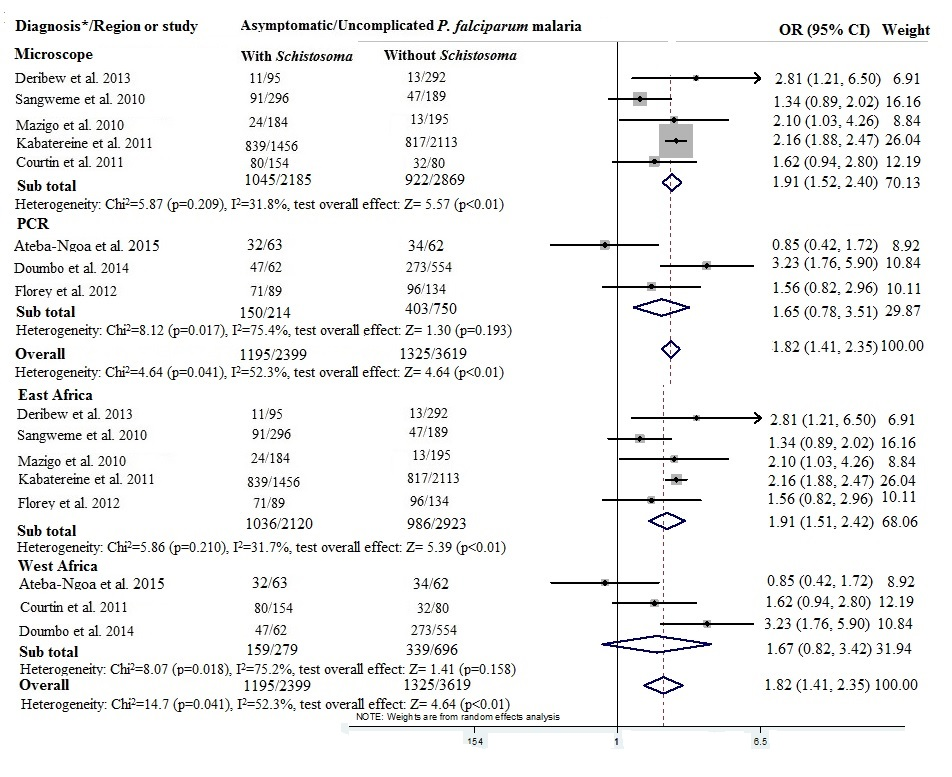

Supplement: S2 Fig — (TIF) [file pntd.0005193.s004.tif]
